# Supplementary material for: Glial type specific regulation of CNS angiogenesis by HIFα-activated different signaling pathways
Source: Nat Commun. 2020 Apr 24;11:2027. doi: 10.1038/s41467-020-15656-4 (PMC7181614; doi:10.1038/s41467-020-15656-4)
Supplement: Supplementary file 3 — Reporting Summary [file 41467_2020_15656_MOESM3_ESM.pdf]

nature research

Corresponding author(s): Fuzheng Guo

Last updated by author(s): Jan 3, 2020

Reporting Summary

Nature Research wishes to improve the reproducibility of the work that we publish. This form provides structure for consistency and transparency in reporting. For further information on Nature Research policies, see Authors & Referees and the Editorial Policy Checklist .

Statistics

For all statistical analyses, confirm that the following items are present in the figure legend, table legend, main text, or Methods section.

n/a	Confirmed	
☐	☒	The exact sample size (n) for each experimental group/condition, given as a discrete number and unit of measurement
☐	☒	A statement on whether measurements were taken from distinct samples or whether the same sample was measured repeatedly
☐	☒	The statistical test(s) used AND whether they are one- or two-sided<br>*Only common tests should be described solely by name; describe more complex techniques in the Methods section.*
☒	☐	A description of all covariates tested
☐	☒	A description of any assumptions or corrections, such as tests of normality and adjustment for multiple comparisons
☒	☐	A full description of the statistical parameters including central tendency (e.g. means) or other basic estimates (e.g. regression coefficient) AND variation (e.g. standard deviation) or associated estimates of uncertainty (e.g. confidence intervals)
☐	☒	For null hypothesis testing, the test statistic (e.g. F, t, r) with confidence intervals, effect sizes, degrees of freedom and P value noted<br>*Give P values as exact values whenever suitable.*
☒	☐	For Bayesian analysis, information on the choice of priors and Markov chain Monte Carlo settings
☒	☐	For hierarchical and complex designs, identification of the appropriate level for tests and full reporting of outcomes
☒	☐	Estimates of effect sizes (e.g. Cohen's d, Pearson's r), indicating how they were calculated

Our web collection on statistics for biologists contains articles on many of the points above.

Software and code

Policy information about availability of computer code

Data collection	Confocal images were obtained using Nikon C1. RT-qPCR were performed on Stragene MxPro-Mx3005P. Protein PAGE seperation was performed using Bio-Rad Mini-Protean Tetra System. Western blot protein transfer was performed using Bio-Rad Tran-Blot Turbo system. Signal development of Western blot was performed using either by Kodak X-OMAT 200A Processor (conventional film) or Li-Cor Odyssey Clx (fluorescence). Western blot signaling quantification was performed using NIH Image J. DNA gel images was obtained using Gel Logic 200 Imaging System.
Data analysis	All quantitative graphs and statistic analyses were generated using GraphPad Prism 8.

For manuscripts utilizing custom algorithms or software that are central to the research but not yet described in published literature, software must be made available to editors/reviewers. We strongly encourage code deposition in a community repository (e.g. GitHub). See the Nature Research guidelines for submitting code & software for further information.

Data

Policy information about availability of data

All manuscripts must include a data availability statement. This statement should provide the following information, where applicable:

- Accession codes, unique identifiers, or web links for publicly available datasets
- A list of figures that have associated raw data
- A description of any restrictions on data availability

The source data underlying Figs. 1b-c, e-m, 2a-k,3b-d, 4c-f, h, j, k, l, 5c-f, 6a-c, f-i, 7a-g, 8c-d, f-g, 9a-b, d-e, g-h, 10b, d, Supplementary Figs. 3b-d, 4c, f-g, 6, 7b, 8a-b, 9b-c, 10a-d, 11b, 12b-c, e-f, 13b are provided as a Source Data file. All other data are available from the corresponding author.

Field-specific reporting

Please select the one below that is the best fit for your research. If you are not sure, read the appropriate sections before making your selection.

- [x] Life sciences
- [ ] Behavioural & social sciences
- [ ] Ecological, evolutionary & environmental sciences

For a reference copy of the document with all sections, see nature.com/documents/nr-reporting-summary-flat.pdf

Life sciences study design

All studies must disclose on these points even when the disclosure is negative.

Sample size	No statistical methods were used for sample size. We used at least three different mice (sexes mixed) throughout the study.
Data exclusions	No data were excluded from analyses in this study.
Replication	All results were replicated in at least two independent experiments
Randomization	N/A
Blinding	The investigators were blinded to the mouse genotype when quantifying data, but not when performing statistical analyses.

Reporting for specific materials, systems and methods

We require information from authors about some types of materials, experimental systems and methods used in many studies. Here, indicate whether each material, system or method listed is relevant to your study. If you are not sure if a list item applies to your research, read the appropriate section before selecting a response.

Materials & experimental systems

n/a	Involved in the study	
☐	☒	Antibodies
☒	☐	Eukaryotic cell lines
☒	☐	Palaeontology
☐	☒	Animals and other organisms
☒	☐	Human research participants
☒	☐	Clinical data

Methods

n/a	Involved in the study	
☒	☐	ChIP-seq
☒	☐	Flow cytometry
☒	☐	MRI-based neuroimaging

Antibodies

Antibodies used

Primary antibodies used for immunohistochemistry: EYFP/GFP (06-896, RRID: AB_310288, 1;500; Millipore), Laminin (L9393, RRID:AB_477163, 1:500, Sigma), Pecam1(sc-1506-R, RRID:AB_831096, 1:100, Santa Cruz Biotechnology), ERG(97249, RRID:AB_2721841, 1:200,Cell signal Technology), PKM2 (4053, RRID:AB_1904096,1:200, Cell signal Technology), Albumin (A90-134A, RRID:AB_67016, 1:200, Bethyl Laboratories), HIF1α (NB100-105,RRID:AB_1001154,1;200, Novus), GFAP (Z0334,RRID:AB_10013382, 1:400, Agilent Technologies), NeuN (MAB377, RRID:AB_2298772,1:200, Millipore), S100β (ab66028,RRID: AB_1142710, 1:200, Abcam) , Sox10 (sc-17342, RRID: AB_2195374, 1:100;Santa Cruz Biotechnology), BrdU (sc-70441, RRID: AB_1119696, 1:100; Santa Cruz Biotechnology), biotin conjugated-Isolectin B4, IB4 (L2140, RRID: AB_2313663, 1:100, Sigma); Active -catenin (05-665, RRID: AB_309887, 1:200, Millipore).

Primary antibodies used for Western blot: beta-actin (Cell signaling, #3700, RRID: AB_2242334, 1:5,000), PKM2 (Cell signaling, #4053, RRID:AB_1904096, 1:2000), HK2 (Cell signaling, #2867, RRID:AB_2232946, 1:2000), MCT1 (Santa Cruz, sc-50325, RRID:AB_2083632, 1:2000), beta-catenin (BD, #610153, RRID: AB_ 397554, 1:1000), Active beta-catenin (Millipore, 05-665, RRID: AB_309887, 1:1000), Axin2 (Prosci, #6163, RRID: AB_10904353, 1:1000), ERG (Cell signaling, #97249, RRID: AB_2721841, 1:2500)

Validation

Primary antibodies were identified by unique RRIDs. The specificity of primary antibody was confirmed by cells, sections, or tissues lacking the target protein

Animals and other organisms

Policy information about studies involving animals; ARRIVE guidelines recommended for reporting animal research

Laboratory animals

All wildtype and transgenic mice were maintained on a C57BL background. Cre transgenic gene was maintained as hemizygote. Both male and female mice were used in the study.

Wild animals	N/A
Field-collected samples	N/A
Ethics oversight	Animal protocols were approved by the IACUC committee at the University of California, Davis

Note that full information on the approval of the study protocol must also be provided in the manuscript.
