# Supplementary material for: Glial type specific regulation of CNS angiogenesis by HIFα-activated different signaling pathways
Source: Nat Commun. 2020 Apr 24;11:2027. doi: 10.1038/s41467-020-15656-4 (PMC7181614; doi:10.1038/s41467-020-15656-4)
Supplement: Supplementary file 2 — Peer Review [file 41467_2020_15656_MOESM2_ESM.pdf]

Reviewers' comments:

Reviewer #1 (Remarks to the Author):

Recent studies have suggested a major role for oligodendrocyte in the regulation of CNS angiogenesis. Specifically, it has been proposed that oligodendrocyte lineage cells promote the proliferation of CNS endothelial cells through a hypoxia inducible factor alpha (HIFa) pathway that is mediated by Wnt but not VEGF signaling. In the current manuscript the authors have reexamined this hypothesis using a variety of genetic approaches. Consistent with earlier studies the authors demonstrate that deletion of HIFa using Cnp-Cre to target oligodendrocyte lineage cells results in a reduction in CNS blood vessel density and endothelial proliferation. Conversely, blocking HIFa degradation results in increased CNS vasculature. The stabilization of HIFa in Cnp+ cells did not however lead to the predicted elevation in Wnt expression, a result that was confirmed using Sox-10 targeting. Furthermore, blocking Wnt signaling in either OPCs or oligodendrocytes did not alter CNS angiogenesis suggesting that Wnt signaling is not a critical component of oligodendrocyte mediated CNS angiogenesis. By contrast to earlier work the authors provide data suggesting the primary downstream signaling pathways from HIFa in OPCs/oligos is mediated by VEGF and that Wnt signaling is largely derived from GFAP+ astrocytes.

Understanding the interplay between glial populations and developing vasculature in the CNS is of significant interest. The data in the current manuscript is extensive and consistent with the interpretation of the authors. There are however a number of issues with the manuscript.
First, while the authors have used some established targeting Cre's to deliver cells specific changes in gene expression there is no data clearly demonstrating the specific cell targeting in the Cre-lox system. It is known that cellular targeting with Cnp and other Cre's may be less specific than originally described. This is even more important where the authors are using inducible constructs and yet the level of induction is poorly defined. Since the data hinges so strongly on cell type localization this would seem to be a critical issue.

Second, the data in Fig 1-3 appear to be largely confirmatory and add little new insights.

Third, the conceptual advance in the manuscript is greatly over exaggerated. What the authors suggest is that different signaling pathways may mediate glial type specific angiogenesis. While this is an advance in our understanding it does not constitute a paradigm shift and the potential relevance in a clinical/pathological situation is not immediately obvious.

Reviewer #2 (Remarks to the Author):

This is an interesting manuscript that addresses the role of oligodendrocyte and astrocyte HIFa-activated signaling in angiogenesis. The data, in general, are clearly presented and convincing. A strength is that most assessments are done multiple ways, adding to the strength of the findings. Some concerns are noted as follows:

1. In multiple experiments the effects of HIFa are determined by genetically disrupting VHL, rather than directly disrupting HIFa. Success of the stabilization of HIFa is assessed by evaluation of its downstream genes and proteins. It would be helpful and more direct to know if deletion of HIFa would similarly not have effects on Wnt/B-catenin activity.

2. Figure 2 D should be improved to better substantiate the assertion that HIF1a is stabilized.

3. In Figure 4 the lack of effect of deletion of VHL is indicated on B-catenin in the Cnp mice, but not in experiments looking at Sox10 or Pdgfra mice. Instead readouts of Wnt/B-catenin signaling are

indicated. It would be more direct to look at B-catenin in these mice as well.

4. Please indicate whether tamoxifen was administered to both the controls as well as the inducible floxed mice.

5. In Figure 2D it does not look as though HIF1a is in the nucleus, as indicated in the legend. Figure 7E should be improved to represent the observation. Figure 9A should be improved to illustrate that EYFP and S100B are co-localized.

6. In the text Supplementary Figure 3 was not discussed.

Reviewer #3 (Remarks to the Author):

The manuscript by Zhang et al, addresses the important question of glio-vascular communication. The current state of knowledge is based on a manuscript published five years ago, which suggested a role of HIF-induced WNT expression in oligodendrocyte progenitors, as the signal regulating in an autocrine manner the inhibition of OL differentiation and in a paracrine manner promoting angiogenesis.
Using a variety of genetic tools, this manuscript challenges the current dogma, and propose that oligodendroglia (progenitors and mature OL) and astroglia cooperate in regulating angiogenesis via the HIF-mediated secretion of VEGFA. There are a number of strengths in this manuscript, including the use of several lines of transgenic mice and a detailed analysis of the angiogenesis phenotype in vivo, in spinal cord and cortex. It is also important that, similar to Yuen et al., the authors demonstrate that stabilization of HIF (due to ablation of VHL either using Cnp-cre or Sox10-cre drivers) results in increased angiogenesis, as indicated by increased PECAM and proliferation of ERG+ endothelial cells (Figs. 2 and 3), while the ablation of HIF function results in decreased proliferation and angiogenesis (Fig.1).

However there are a number of MAJOR CONCERNS that need to be addressed in order to strengthen the overall message:

1. The current study challenges HIF-regulation of WNT as paracrine mechanism of regulation of angiogenesis, based on at least three lines of evidence in CnpCreVhlfl/fl mice : similar levels of Wnt7a and its target genes in spinal cord and forebrain tissues at distinct time points and lack of activity using a Wnt reporter transgenic mouse. Importantly, as it was previously described that Wnt7a expression declines as OPC differentiate , the authors also report similar gene expression also in tissue from two additional lines Sox10CreVhlfl/fl and PdgfraCreERT2Vhlfl/fl . While the data are convincing, one would argue that a more direct approach is needed to challenge the mechanism of autocrine regulation. Assessing the levels of Wnt7a and its target genes as well as Hk2 (as control for HIF activity) in progenitor cells either directly sorted from the brain and spinal cord or cultured from the neonatal brain is truly essential.

2. Part of the rationale in support of previously suggested paracrine regulation of angiogenesis by WNT-secretion from OPC, was based on the results on pharmacological inhibitors of the palmitoyl acyltransferase Porcupine. This study adopts a genetic approach targeting a distinct secretory pathway involving Wntless (Wls). Since these two secretory pathways may differentially impact WNT secretion, it is very important for the authors to provide clear evidence of impaired WNT secretion in their triple mutant. One could achieve this, for instance, by measuring WNT levels in the culture medium of OPC isolated from Sox10CreERT2Vhlfl/fl Wlsfl/fl.. Without this critical piece of information , is difficult to assess whether Wnt secretion was truly impaired in these mice, and therefore any interpretation of the mouse phenotype should be carefully considered.

3. While the demonstration that VEGFA secretion by oligodendroglia is very convincing, one would want to understand why the authors would test the hypothesis of astroglial secreted VEFGA as regulator of angiogenesis, by analyzing p60 tissue. Evaluation of the effect on blood vessels during the early postnatal period is absolutely necessary.

Minor points:

1. it would be important to show that CnpCre behaves like no-Cre control, at least in one supplemental figure
2. Fig. 3 B, D, E have typos in the Y axis

Response to Reviewers
Zhang, Kim et al., NCOMMS-19-12703

We thank all three reviewers for their insightful comments, which have been addressed as outlined below

Reviewer #1 (Remarks to the Author):

Recent studies have suggested a major role for oligodendrocyte in the regulation of CNS angiogenesis. Specifically, it has been proposed that oligodendrocyte lineage cells promote the proliferation of CNS endothelial cells through a hypoxia inducible factor alpha (HIFa) pathway that is mediated by Wnt but not VEGF signaling. In the current manuscript the authors have reexamined this hypothesis using a variety of genetic approaches. Consistent with earlier studies the authors demonstrate that deletion of HIFa using Cnp-Cre to target oligodendrocyte lineage cells results in a reduction in CNS blood vessel density and endothelial proliferation. Conversely, blocking HIFa degradation results in increased CNS vasculature. The stabilization of HIFa in Cnp+ cells did not however lead to the predicted elevation in Wnt expression, a result that was confirmed using Sox-10 targeting. Furthermore, blocking Wnt signaling in either OPCs or oligodendrocytes did not alter CNS angiogenesis suggesting that Wnt signaling is not a critical component of oligodendrocyte mediated CNS angiogenesis. By contrast to earlier work the authors provide data suggesting the primary downstream signaling pathways from HIFa in OPCs/oligos is mediated by VEGF and that Wnt signaling is largely derived from GFAP+ astrocytes.

Comment #1: *"…while the authors have used some established targeting Cre's to deliver cells specific changes in gene expression there is no data clearly demonstrating the specific cell targeting in the Cre-lox system. It is known that cellular targeting with Cnp and other Cre's may be less specific than originally described. This is even more important where the authors are using inducible constructs and yet the level of induction is poorly defined. Since the data hinges so strongly on cell type localization this would seem to be a critical issue".*

Responses: In the revised manuscript, we have included experimental data showing the specificity and recombination efficiency of the inducible Cre transgenic strains that we used in the current study. Please see Supplementary Fig. 7 (*Pdgfrα-CreER^{T2}* strain), Supplementary Fig. 11 (*Sox10-CreER^{T2}*), and Supplementary Fig. 13 (*Aldh1l1-CreER^{T2}*).

We agree with the reviewer that *Cnp-Cre* is less specific than originally described. Our previous study (Lang et al., 2013 J Neurosci. PMCID: PMC3711764) reported that, in addition to originally descried specificity in oligodendroglial lineage cells, *Cnp-Cre* is also active in a subset of early neural progenitor cells, which was later confirmed in a recent study (Tognatta et al., 2017 Glia, PMCID: PMC6813834). The conclusions drawn from *Cnp-Cre* transgenic strain were strengthened by analyzing the constitutive Sox10-Cre strain, which was shown a much greater specificity in oligodendroglial lineage cells (Yuen et al., 2014 Cell, PMCID: PMC4149873). More importantly, our conclusions were also supported by employing tamoxifen-inducible *Sox10-CreER^{T2}* and *Pdgfrα-CreER^{T2}*, both of which circumvent the concerns of possible ectopic Cre activity in early neural progenitor cells.

Comment #2: *"…the data in Fig 1-3 appear to be largely confirmatory and add little new insights"*.

Responses: This is a great point! The aim of Figs. 1-3 in the initial submission was to demonstrate the CNS region-independent regulation of angiogenesis by oligodendroglial HIFα (brain vs spinal cord) since recent study only analyzed brain angiogenesis. In the revised manuscript, we have clarified this point that oligodendroglial regulation of angiogenesis appears to be a common phenomenon throughout the CNS. Furthermore, the data of initial Fig. 1-3, which were collected from different transgenic mice, together with recent published data (Yuen et al., 2014 Cell, PMCID: PMC4149873) provide sound scientific premise on which our mechanistic study was built.

We agree with the reviewer that the first three figures appear to be largely confirmatory. Accordingly, we have removed most of the panels into supplemental figures and

integrated the key data of initial Fig. 1-3 supporting the CNS region-independent angiogenic regulation into the revised figure 1.

Comment #3: "…*the conceptual advance in the manuscript is greatly over exaggerated. What the authors suggest is that different signaling pathways may mediate glial type specific angiogenesis. While this is an advance in our understanding it does not constitute a paradigm shift and the potential relevance in a clinical/pathological situation is not immediately obvious."*

Responses: A great point. We thank the reviewer for this constructive comment. We have avoided doing this. In brief, we have changed the manuscript title to "Glial type specific regulation of CNS angiogenesis by HIFα-activated different signaling pathways". In the meantime, we have removed the phrases of "conceptual shift" throughout the manuscript.

Reviewer #2 (Remarks to the Author):

This is an interesting manuscript that addresses the role of oligodendrocyte and astrocyte HIFa-activated signaling in angiogenesis. The data, in general, are clearly presented and convincing. A strength is that most assessments are done multiple ways, adding to the strength of the findings. Some concerns are noted as follows:

We thank the reviewer for the kind comments.

Comment #1: *"…It would be helpful and more direct to know if deletion of HIFa would similarly not have effects on Wnt/B-catenin activity"*

Responses: We have included additional analysis of Wnt/beta-catenin activity in *Cnp-Cre:HIFa* cKO mutants and *Pdgfra-CreERT2:HIFa* cKO mutants. In brief, we found that deletion of HIFa had no detectable effects on Wnt/beta-catenin activity. Please see Supplementary Figure 10 for details.

Comment #2: *"…Figure 2 D should be improved to better substantiate the assertion that HIF1a is stabilized."*

Responses: We have replaced Figure 2D with more convincing confocal images showing HIF1a stabilization in VHL-deficient oligodendrocytes. Please see Supplementary Figure 4d for details.

Comment #3: *"… In Figure 4 the lack of effect of deletion of VHL is indicated on B-catenin in the Cnp mice, but not in experiments looking at Sox10 or Pdgfra mice. Instead readouts of Wnt/B-catenin signaling are indicated. It would be more direct to look at B-catenin in these mice as well...*"

Responses: We thank the reviewer for this comment. We have included Western blot data of active beta-catenin assessed in *Sox10-Cre:VHL* cKO and *Pdgfra-CreER*^{T2}*:VHL* cKO mice. Please see Supplementary Figure 6 and 8 for details

Comment #4: *"…Please indicate whether tamoxifen was administered to both the controls as well as the inducible floxed mice…"*

Responses: We have added the description of "tamoxifen was administered to both the controls and the inducible floxed mice in all experiments involving inducible Cre-LoxP approach".

Comment #5: "*…. In Figure 2D it does not look as though HIF1a is in the nucleus, as indicated in the legend. Figure 7E should be improved to represent the observation. Figure 9A should be improved to illustrate that EYFP and S100B are co-localized.*"

Responses: We thank the reviewer for those comments. Figure 2D has been corrected as indicated in Comment #2. We have replaced Figure 7E with more convincing images showing Vegfa mRNA signals were elevated in *Plp* mRNA-positive cells in *Cnp-Cre:VHL* cKO mice (please see Figure 6e1-e2). Figure 9A has been replaced with the confocal images showing clear colocalization of EYFP and astrocytic marker S100beta (please see Figure 8a)

Comment #6: *"… In the text Supplementary Figure 3 was not discussed…"*

Responses: We apologize for this oversight. This supplemental figure (Supplemental Figure 14 in the revised manuscript) is a diagram summarizing the major findings in our

study. We have discussed this figure in the revised Discussion section.

Reviewer #3 (Remarks to the Author):

The manuscript by Zhang et al, addresses the important question of glio-vascular communication. The current state of knowledge is based on a manuscript published five years ago, which suggested a role of HIF-induced WNT expression in oligodendrocyte progenitors, as the signal regulating in an autocrine manner the inhibition of OL differentiation and in a paracrine manner promoting angiogenesis. Using a variety of genetic tools, this manuscript challenges the current dogma, and propose that oligodendroglia (progenitors and mature OL) and astroglia cooperate in regulating angiogenesis via the HIF-mediated secretion of VEGFA. There are a number of strengths in this manuscript, including the use of several lines of transgenic mice and a detailed analysis of the angiogenesis phenotype in vivo, in spinal cord and cortex. It is also important that, similar to Yuen et al., the authors demonstrate that stabilization of HIF (due to ablation of VHL either using Cnp-cre or Sox10-cre drivers) results in increased angiogenesis, as indicated by increased PECAM and proliferation of ERG+ endothelial cells (Figs. 2 and 3), while the ablation of HIF function results in decreased proliferation and angiogenesis (Fig.1). However there are a number of MAJOR CONCERNS that need to be addressed in order to strengthen the overall message.

We thank the reviewer for the positive and kind comments. We have performed additional experiments and analyses to address the three major concerns.

Comment #1: *"…While the data are convincing, one would argue that a more direct approach is needed to challenge the mechanism of autocrine regulation. Assessing the levels of Wnt7a and its target genes as well as Hk2 (as control for HIF activity) in progenitor cells (OPCs) either directly sorted from the brain and spinal cord or cultured from the neonatal brain is truly essential."*

Responses: This is an excellent point. The authors thank the reviewer for this critique. We cultured primary OPCs isolated from the neonatal brain of Sox10-Cre, $Vhl^{fl/fl}$ and non-Cre littermate controls. Our data demonstrated that the levels of Wnt7a and Wnt target genes Axin2 and Naked1 were indistinguishable in brain OPCs from *Sox10-Cre,*

Vhl$^{fl/fl}$ and non-Cre littermates, which was in stark contrast to the significant upregulation of HIFα target genes *Hk2, Ldha, Glut1*, and *Pkm2*. Our results indicate that genetically stabilizing HIFα in OPCs does not activate Wnt7a, nor perturbs autocrine Wnt signaling pathway. Please also see Figure 2j-k in the revised manuscript for details.

Comment #2: *"…This study adopts a genetic approach targeting a distinct secretory pathway involving Wntless (Wls). Since these two secretory pathways (note: the other involving palmitoyl acyltransferase Porcupine) may differentially impact WNT secretion, it is very important for the authors to provide clear evidence of impaired WNT secretion in their triple mutant … Without this critical piece of information, it is difficult to assess whether Wnt secretion was truly impaired in these mice, and therefore any interpretation of the mouse phenotype should be carefully considered".*

Responses: To provide direct evidence of impaired Wnt secretion in WLS-deficient OPCs, we took advantage of enforced Wnt7a expression with simultaneous WLS knockdown in primary OPCs (see Methods). Our results clearly demonstrated that the level of Wnt7a protein secreted from Wnt7a-expressing OPCs (quantified by Wnt7a ELISA of the culture media) was significantly diminished in WLS-deficient OPCs, suggesting that WLS is required for Wnt secretion from OPCs. Please see Figure 3 in the revised manuscript for details.

As indicated in Comment #1 and responses, HIFα stabilization did not impair Wnt7a and Wnt signaling in OPCs. Figure 3c showed that primary OPCs did not secrete substantial amount of Wnt7a, evidenced by the similar levels of Wnt7a protein in the growth media (GM) with or without OPCs in the dish. These data justifies the usage of enforced Wnt7a-expressing OPCs in our experiment.

Comment #3: *"…While the demonstration that VEGFA secretion by oligodendroglia is very convincing, one would want to understand why the authors would test the hypothesis of astroglial secreted VEFGA (note: Wnt secretion) as regulator of angiogenesis, by analyzing p60 (note: p30) tissue. Evaluation of the effect on blood vessels during the early postnatal period is absolutely necessary".*

Responses: In our initial submission, we analyzed *mGfap-Cre, $Vhl^{fl/fl}$, $Wls^{fl/fl}$* mutants at p30 because of the poor recombination rate in the early postnatal CNS of mGfap-Cre mice (please see Supplementary Fig. 12 for details). To address this concern, we employed *Aldh1l1-$CreER^{T2}$* transgenic mice, in which we showed a greater than 90% of recombination rate in astrocytes at the early postnatal age P8 (tamoxifen treatment at P1, P2, and P3) (please see Supplementary Fig. 13).

Consistent with the data derived from p30 *mGfap-Cre, $Vhl^{fl/fl}$, $Wls^{fl/fl}$* mutants, we found that blocking Wnt secretion from early postnatal astrocytes significantly reduced HIFα-elicited elevation of CNS angiogenesis in *Aldh1l1-$CreER^{T2}$, $Vhl^{fl/fl}$, $Wls^{fl/fl}$* mutants at P8 compared with non-Cre controls (tamoxifen treatment both mutant and non-Cre Ctrl at P1, P2, and P3). These data strengthen the conclusion in our initial submission that astroglial HIFα acts through Wnt signaling to regulate angiogenesis in the early postnatal CNS.

Comment #4: *"…it would be important to show that CnpCre behaves like no-Cre control, at least in one supplemental figure…"*

Responses: We analyzed CNS angiogenesis and motor function of non-Cre control and CnpCre transgenic mice and found no difference in these aspects. Please see Supplementary Figure 3 for details.

Comment #5: *"…Fig. 3 B, D, E have typos in the Y axis (X- spinal cord)"*

Responses: We have corrected these typos in the revised manuscript.

We thank the reviewers for their insightful critiques in improving our study!

Best regards,e

Sheng Zhang, PhD

Bokyung Kim, PhD

Fuzheng Guo, PhD

REVIEWERS' COMMENTS:

Reviewer #1 (Remarks to the Author):

In this revised manuscript the authors have addressed many of the concerns raised in the previous reviews and the paper is significantly stronger as a result. The addition of new data showing the efficiency of the genetic approaches and the analysis of direct interactions of HIFa in cells of the oligodendrocyte lineage. The paper is more balanced in its presentation and the revisions and new data make a more compelling case that stabilization of HIFa in oligodendrocytes activates VEGF but does not modulate Wnt signaling. This is an important observation that provided novel insights into the role of glial in CNS angiogenesis.

There are only minor issues that require attention.
Lines 80-87. It would be good to include the age at which analysis was done. It is in the legend but should be in the results.
Lines 161-170 The revised text could be improved particularly the sentence "We expressed Wnt7a…."

Reviewer #3 (Remarks to the Author):

The authors have provided compelling evidence to address all the previous concerns.

The inclusion of new data in Fig2 has nicely addressed previous concerns as it provides convincing evidence against the concept of HIF1a- dependent secretion of WNT7a and its targets in OPC.

The data in Figure 3 further show the involvement of the WLS secretory pathway in OPC induced to express Wnt7A (as they do not express detectable levels in physiological conditions.

Finally the inclusion of early postnatal data on Aldh1l1-Cre Vhlfl/fl mice and on triple transgenic Aldh1l1-Cre Vhlfl/fl Wls fl/fl nicely support the authors interpretation

Response to Reviewers
Zhang, Kim et al., NCOMMS-19-12703A

We thank reviewers for their insightful comments, which have been addressed as outlined below.

Reviewer #1 (Remarks to the Author):

In this revised manuscript the authors have addressed many of the concerns raised in the previous reviews and the paper is significantly stronger as a result. The addition of new data showing the efficiency of the genetic approaches and the analysis of direct interactions of HIFa in cells of the oligodendrocyte lineage. The paper is more balanced in its presentation and the revisions and new data make a more compelling case that stabilization of HIFa in oligodendrocytes activates VEGF but does not modulate Wnt signaling. This is an important observation that provided novel insights into the role of glial in CNS angiogenesis. There are only minor issues that require attention.

Comment #1: *Lines 80-87. It would be good to include the age at which analysis was done. It is in the legend but should be in the results.*

Responses: Thank the reviewer for the suggestion. We have added the ages in the Cnp-Cre transgene and HIF1α/HIF2α double cKO mice.

Comment #2: *Lines 161-170 The revised text could be improved particularly the sentence "We expressed Wnt7a…."*

Responses: We have revised that sentence as "Because Wnt7a has been shown as one of the major Wnt ligand genes expressed in OPCs at the mRNA level, we overexpressed Wnt7a in primary OPCs."

Reviewer #3 (Remarks to the Author):

The authors have provided compelling evidence to address all the previous concerns. The inclusion of new data in Fig2 has nicely addressed previous concerns as it provides

convincing evidence against the concept of HIF1a- dependent secretion of WNT7a and its targets in OPC.

The data in Figure 3 further show the involvement of the WLS secretory pathway in OPC induced to express Wnt7A (as they do not express detectable levels in physiological conditions.

Finally, the inclusion of early postnatal data on Aldh1l1-Cre Vhlfl/fl mice and on triple transgenic Aldh1l1-Cre Vhlfl/fl Wls fl/fl nicely support the authors interpretation

Responses: We thank you for the positive feedback from the reviewer.

Best regards,

Sheng Zhang, PhD

Bokyung Kim, PhD

Fuzheng Guo, PhD
